# General Management of Female Sexual Dysfunction for Urologists

**DOI:** 10.5152/tud.2021.20588

**Published:** 2023-01-01

**Authors:** Johannes Bitzer

**Affiliations:** 1 Department of Obstetrics and Gynecology, University Hospital Basel Ringgold Standard Institution, Basel, Switzerland

**Keywords:** Biopsychosocial model, female sexual dysfunction, multidisciplinary approach

## Abstract

Female sexual dysfunctions are grouped into desire, arousal, orgasmic, and sexual pain disorders according to international classification systems. The disorders frequently overlap and coexist, and the pathogenesis is in most cases due to an interaction of biological (body), psychological (mind), and sociocultural (environment) factors. Typical medical conditions are hormonal changes, depression, and drug treatment. Urological problems having a negative impact are incontinence, prolapse, and overactive bladder. Frequent psychological factors are lack of knowledge about the body, traumatic or negative experiences, and performance anxiety. Relationship factors include conflicts and difficulties in communication. The prevalence of the disorders varies over age groups. In adolescents, pain and orgasmic disorders are predominant, and later in life, arousal difficulties may arise accompanied by low desire. Based on the biopsychosocial concept, therapies frequently include concomitant medical and psychotherapeutic interventions in a multidisciplinary approach.

**Main Points**

- Female sexual dysfunctions are grouped into desire, arousal, orgasmic, and sexual pain disorders.
- The disorders frequently overlap and coexist, and the pathogenesis is in most cases due to an interaction of biological (body), psychological (mind), and sociocultural (environment) factors.
- Based on the biopsychosocial concept, therapies frequently include concomitant medical and psychotherapeutic interventions in a multidisciplinary approach.

## Introduction and Definitions

Based on the model of the human sexual response cycle described by Masters and Johnson and H S Kaplan, Female Sexual Dysfunctions (FSD) can be classified into 4 groups based on the different components of the response cycle.^1–4^

## Sexual Desire Disorder

### Hypoactive Sexual Desire Disorder

Persistent or recurrent deficiency (or absence) of sexual fantasies/thoughts and/or desire for or receptivity to sexual activity, which *causes personal distress.*

### Sexual Aversion Disorder

Persistent or recurrent phobic aversion to and avoidance of sexual contact with a sexual partner, *which causes distress.*

## Arousal Disorder

Persistent or recurrent inability to attain or maintain sufficient sexual excitement *causing personal distress*. It may be experienced as a lack of subjective excitement or genital (lubrication/ swelling) or other somatic responses.

## Orgasmic Disorder

Persistent or recurrent difficulty, delay in or absence of attaining orgasm following sufficient sexual stimulation and arousal, *which causes personal distress.*

## Sexual Pain Disorders

### Dyspareunia

Recurrent or persistent genital pain associated with sexual intercourse, *which causes personal distress.*

### Vaginismus

Recurrent or persistent involuntary spasm of the musculature of the lower third of the vagina that interferes with vaginal penetration, *which causes personal distress.*

There is an ongoing discussion regarding the available evidence to distinguish between desire and arousal and whether vaginismus and dyspareunia can be distinguished.

In the *Diagnostic and Statistical Manual of Mental Disorders (DSM-V)*, desire and arousal disorders are combined into the category female sexual interest/arousal disorder.

Dyspareunia and vaginismus are combined into genito-pelvic pain/ penetration disorder.^5,6^

## The Biopsychosocial Model of Understanding Female Sexual Dysfunction

Sexual health and sexual dysfunction are the result of a complex interaction of biological, psychological, interpersonal, and social factors. These factors interact with each other and can have a differential impact on the female sexual function, causing dysfunction.^7-9^

## Biological Contributing Factors

- Chronic diseases
  - Cancer, cardiovascular, and metabolic psychiatric diseases
  - Diseases can impact sexual health in the following ways:
  - tissue damage of the organs involved in the sexual response
  - damage to the neuromuscular and neurovascular system
  - influencing the endocrine response
  - impairment of the brain neurotransmitter responses
- Endocrine changes
  - Postpartum
    - Low estrogen, prolactin contributing to low desire, and painful intercourse
  - Menopause
    - Estrogen fluctuation, low estrogen, and testosterone insufficiency
    - Contributing to low desire and arousal difficulties
  - Diabetes
  - Vascular changes in the genital organs of men and women can contribute to arousal problem (erectile dysfunction and arousal disorder in women)
- Medications and recreational drugs
  - Drugs influencing the peripheral response, such as antihypertensives
  - Antidepressants having an impact on serotonin central action contributing to low desire
  - Antihormones having an impact on the genital organs and on the brain regulation of the sexual response

## Individual Psychological Factors

- Immediate factors
  - Sexual anxiety (not knowing what will happen, lack of knowledge)
  - Performance anxiety
  - Fear of getting hurt
- Personality traits
  - Lack of self esteem
  - Body image problem
- Biographic factors
  - Early neglect
  - Sexual abuse
  - Later traumatic experiences
  - Separation, humiliation
  - Sexual violence
  - Lack of sexual education

## Relationship Factors

- Sexual dysfunction partner
  - Erectile dysfunction
  - Premature ejaculation
- Conflicts
  - Jealousy and mistrust
  - Differences in needs
  - Imbalance of giving and taking
  - Inability to compromise and forgive
- Routine
  - Loss of tension
  - Loss of seduction and fantasy
- Lack of communication
  - Avoiding to talk about feelings
  - Fear of hurting the partner
  - Hidden stories
  - Third-party involvement
  - Internet pornography

## Sociocultural and Economic Factors

- General living conditions
- Social distress
- Sociocultural norms

## Treatment of Sexual Dysfunction and Sexual Problems

Owing to the frequently complex pathogenesis, treatment strategies also need to follow a biopsychosocial and, frequently, multidisciplinary approach.

The first step is always basic counseling.^10^

## Basic Counseling

Basic counseling has several components, which have proven therapeutic effects.

Emotional relief

- Give the patient, the opportunity to talk about his/her own sexuality
- Listen actively
- Patient feels accepted and understood

Education and empowerment

- Inform about the reality of human sexuality
- Put the variety of personal experiences into perspective
- Frequency of problems
- Differences between female and male sexuality

Dispel myths about male and female sexuality represented in social media about normal sexuality with different new norms and false information about “good sex.”

“It rocks you and makes you feel like in heaven.”

- Sex is not penis in vagina, the usual program, but there is a large variety of sexual expression and preferences
- The pleasure of nongenital sex
- The use of sex toys is not pornographic
- Having sexual problems means that love has died

The second step is establishing a treatment plan based on the understanding of the contributing factors (see above).

## Treatment Options and Strategies: An Overview^11-13^ Medical Treatment Options

- Examples
  - Treatment of endometriosis in sexual pain disorder
    - Surgical and drug treatment
  - Adaptation of drug treatment in cancer patients
    - Aromatase inhibitors
  - Change of antihypertensives
    - Antihypertensive without negative impact on peripheral vascular function
  - Change of antidepressants
    - Change from tricyclics to Selective Serotonin Reuptake Inhibitors
  - Endocrine treatment
    - Estrogen: The large majority of studies show positive effects of estrogen therapy on different aspects of female sexual function and sexual satisfaction, including desire, arousal, and treatment of sexual pain due to vulvovaginal atrophy.
    - Testosterone and tibolone: Androgens play a role in sexual desire, arousal, orgasm, and satisfaction.
  - PDE-5 inhibitors for erectile dysfunction in men; arousal difficulties in diabetic women
  - Antibiotics for infections causing painful ejaculation in men
  - Dapoxetine for premature ejaculation
  - Centrally acting drugs like flibanserin for low desire in women

## Psychotherapeutic and Psychosexual Interventions (Individual)

- Examples
  - Body awareness methods for orgasmic and arousal difficulties related to insecurity, lack of knowledge about one’s own body
    - Masturbation exercises
    - Therapy sexocorporelle
  - Physiotherapy for sexual pain disorders related to pelvic floor dysfunction
  - Cognitive behavioral interventions for dysfunctions related to the following:
    - irrational beliefs and thoughts leading to negative emotions by becoming aware of these patterns and actively changing perspectives and way of thinking
    - distress and performance anxiety by learning relaxation and coping techniques
  - Psychodynamic focal therapy for dysfunctions related to feelings of guilt, shame, and internal conflicts between sexual needs and sexual norms:
    - making them aware of this dynamics and helping the patient to find a solution based on integration of both parts of his or her personality
  - Mindfulness
- Mindfulness can be defined as a state of present-moment, nonjudgmental awareness
  - Regulation of attention to keep it focused on the immediate experience.
  - Approaching one’s experience with curiosity, openness, and acceptance.
  - The here and now should be observed and experienced with all senses, thoughts and feelings should be perceived without judgment but with an open mind. Thus, every sexual experience is unique.

## Couple-Oriented Interventions

- Examples
  - Communication training for dysfunctions based on dysfunctional communication
    - Listening to each other (facts, emotions, relationship messages, etc.)
    - How to communicate yes and no
    - How to communicate without hurting each other
    - How to give feedback
    - How to negotiate
  - Sensate focus (Masters and Johnson)
    - Masters and Johnsons developed a therapeutic concept, which combined different elements of body awareness, focal psychotherapy, and systemic therapy into one approach.
    - The concept supports couples in “relearning the sexual encounter” by dividing the intercourse into different phases, which allow both partners to observe and communicate about their physical and emotional reactions, thus creating the conditions of an enjoyable sexual encounter without being under the pressure of success.

## Treatment for Specific Dysfunctions Sexual Desire Disorder

### Medical Therapy:

Estrogens, androgens, and tibolone

- Hormone therapy in women with sex hormone deficiency, for example, during menopausal transition or in those women showing signs of Androgen Insufficiency

Addressing co-morbid depression

- Depression is a frequent comorbidity and needs treatment eventually by adjustment of dosages or types of psychotropic drugs

### Psychosexual Therapy

Individual

- Building on previous positive experiences and models of satisfying sexuality
- Addressing negative experiences
- Helping patients to find out what they really want in their sexual life

Couple therapy

- Sensate focus (see above)

## Arousal Disorder

### Medical Therapy

Hormonal therapy (see therapies above)

- Local estrogen therapy to improve vaginal tissue and vaginal blood flow

Blood vessel dilators

- PDE-5 inhibitors have proven to be effective in women with diabetes

### Nonmedical Treatments

Clitoral therapy device

- A Food and Drug Administration approved device such as a vibrator
- Physiotherapy of the pelvic floor

Lifestyle changes

- Physical activities

### Psychosexual Therapy

- Body awareness methods (see above)

## Orgasmic Disorders

*Medical therapy*

- Optimize hormones
- Adjustment of orgasm-inhibiting medications

*Physiotherapy of the pelvic floor*

*Psychosexual therapy*

- Body awareness methods

## Sexual Pain Disorder

*Medical treatment*

- Specific treatment for underlying disease
- Superficial dyspareunia (e.g., vulvovaginal infections and dermatologic diseases)
- Deep dyspareunia (e.g., endometriosis, adhesions, and irritable bowel syndrome)
- Normalize vaginal tropism and pH (by local hormonal treatment)
- Use of moisturizers

*Physiotherapy of pelvic floor*

- Combined with imagination and relaxation techniques

*Psychosexual single and couple therapy*

Mindfulness

- Sensate focus
- Communication training to improve understanding of adequate stimulation

*Analgesic therapy*

- Systemic: NSARs, antidepressants, and anticonvulsants (neuropathic pain)
- Local: Cortisone and injection of anesthetics
- Surgical interventions (vestibulectomy)
